# Factors associated with severe disease and in-hospital mortality in children with non-HIV-associated *Pneumocystis jirovecii* pneumonia: a single-center retrospective cohort study

**DOI:** 10.3389/fcimb.2026.1937817

**Published:** 2026-10-12

**Authors:** Junjie Ning, Dan Chen, Zhidong Yu, Fajuan Tang, Lina Qiao

**Affiliations:** 1 Department of Scientific Research, Zigong First People's Hospital, Zigong, Sichuan, China; 2 Department of Pediatrics, West China Second University Hospital, Sichuan University, Chengdu, Sichuan, China; 3 Department of Pediatrics, Meishan People’s Hospital, Meishan, Sichuan, China; 4 Department of Pediatrics, Zigong First People’s Hospital, Zigong, Sichuan, China

**Keywords:** children, in-hospital mortality, lactate dehydrogenase, non-HIV-associated, Pneumocystis jirovecii pneumonia, severe disease

## Abstract

**Objective:**

To identify factors associated with severe *Pneumocystis jirovecii* pneumonia (PJP) and in-hospital mortality and evaluate their ability to discriminate severe PJP in children without human immunodeficiency virus (HIV) infection.

**Methods:**

This single-center retrospective cohort study included 61 children with non-HIV-associated PJP admitted between February 2022 and June 2025, including 31 with non-severe and 30 with severe PJP. Univariable and multivariable Firth penalized logistic regression identified factors associated with severe PJP, and receiver operating characteristic curves assessed discrimination. A restricted Firth logistic model explored in-hospital mortality among severe cases.

**Results:**

Thirty children (49.2%) had severe PJP, and nine (14.8%) died during hospitalization; all deaths occurred in the severe group. Compared with the non-severe group, the severe group had more frequent primary immunodeficiency, fever, dyspnea, and cytomegalovirus (CMV) coinfection; higher D-dimer, lactate dehydrogenase (LDH), procalcitonin, and CT severity scores; and lower albumin, PaO_2_/FiO_2_, and CD4^+^/CD8^+^ T-cell ratios (all P<0.05). In the primary multivariable model, LDH (per 100-U/L increase: adjusted odds ratio [aOR]=1.532, 95% confidence interval [CI]: 1.061–2.374; P = 0.021) and CMV coinfection (aOR=5.164, 95% CI: 1.099–28.048; P = 0.037) were associated with severe PJP. LDH remained significant in the sensitivity analysis, whereas CMV coinfection did not. LDH discriminated severe from non-severe PJP with an area under the curve of 0.868 (95% CI: 0.776–0.960); the optimal cutoff was 423.0 U/L, with 90.0% sensitivity and 71.0% specificity. The combined model had an AUC of 0.878 (95% CI: 0.791–0.966), without significant improvement over LDH alone (P = 0.701). Among severe cases, non-survivors had more frequent malnutrition and CMV coinfection, higher LDH and procalcitonin, and lower neutrophil counts, albumin, PaO_2_/FiO_2_, and CD4^+^/CD8^+^ T-cell ratios (all P<0.05). In the exploratory mortality model, LDH (per 100-U/L increase: aOR=1.654, 95% CI: 1.080–4.271; P = 0.018) and albumin (per 1-g/L increase: aOR=0.605, 95% CI: 0.251–0.878; P = 0.004) were associated with in-hospital mortality.

**Conclusions:**

Elevated LDH was consistently associated with severe PJP and showed good discrimination. CMV coinfection was associated with severe PJP only in the primary model and provided no significant incremental discrimination. Elevated LDH and decreased albumin may be associated with in-hospital mortality. Larger external cohorts are needed to validate these findings.

## 1 Introduction

*Pneumocystis jirovecii* pneumonia (PJP) is a potentially life-threatening opportunistic infection caused by *Pneumocystis jirovecii* (*P. jirovecii*) and occurs predominantly in individuals with impaired cell-mediated immunity. With the widespread use of effective antiretroviral therapy and routine pharmacological prophylaxis, the incidence of HIV-associated PJP has declined substantially. However, the growing populations of patients with malignancies, solid-organ or hematopoietic stem cell transplantation, primary immunodeficiencies, and exposure to corticosteroids or other immunosuppressive therapies have made non-HIV-associated PJP an increasingly important clinical concern (Xue et al., 2023). It has been estimated that, among more than 400, 000 PJP cases occurring worldwide each year, approximately 60% occur in individuals without HIV infection (Rhoads et al., 2024).

Previous studies have shown that, compared with HIV-associated PJP, non-HIV-associated PJP generally has a more acute onset and a more rapidly progressive clinical course and is associated with higher risk of respiratory failure, mechanical ventilation, and in-hospital mortality (Cillóniz et al., 2019; Rego de Figueiredo et al., 2019). In addition, nearly 50% of patients with PJP may develop acute lung injury, and among survivors, patients with a history of PJP may be at greater risk of long-term pulmonary functional impairment than survivors of bacterial pneumonia (Maschmeyer et al., 2016). Therefore, early identification of patients at high risk of developing severe disease or adverse outcomes is clinically important. However, the early diagnosis and risk assessment of non-HIV-associated PJP remains challenging. The clinical and radiological manifestations of PJP are nonspecific and may resemble those of other pulmonary infections or noninfectious lung diseases. Furthermore, conventional microscopic examination has limited sensitivity, particularly in non-HIV patients with relatively low organism burden, resulting in a greater likelihood of false-negative findings. Although highly sensitive molecular methods, such as polymerase chain reaction, have improved the detection of *P. jirovecii*, a positive result does not necessarily indicate active PJP, and active infection must be distinguished from respiratory tract colonization (Rojas et al., 2017; Sun et al., 2025). Therefore, identifying susceptibility factors for PJP may help assess the likelihood of active disease and support clinical diagnosis.

Several risk factors for the development of PJP have been identified, including lymphopenia, corticosteroid use (peak doses <0.5 or ≥0.5 mg/kg/day), and impaired CD4^+^ T-cell immune reconstitution (Lécuyer et al., 2024; Huang et al., 2026; Hulot et al., 2026). In addition, elevated lactate dehydrogenase (LDH) levels and cytomegalovirus (CMV) coinfection may be associated with disease severity or adverse outcomes in patients with non-HIV-associated PJP (Wang et al., 2021; Li et al., 2026). However, previous studies have primarily focused on adults receiving chemotherapy for malignancies, recipients of solid-organ or hematopoietic stem cell transplantation, and patients receiving immunosuppressive therapy for autoimmune diseases. Whether these findings can be directly extrapolated to children remains uncertain. Clinical studies of non-HIV-associated PJP in children remain limited, and factors associated with disease severity and prognosis after diagnosis are not yet fully understood.

Therefore, this single-center retrospective cohort study aimed to characterize the clinical features and in-hospital outcomes of non-HIV-infected children with PJP, compare the clinical characteristics of children with severe and non-severe PJP, and investigate factors associated with severe disease. Among children with severe PJP, we further compared the clinical characteristics of survivors and non-survivors and explored factors associated with in-hospital mortality, with the aim of informing early risk stratification and clinical management of pediatric PJP.

## 2 Materials and methods

### 2.1 Study population

This single-center retrospective cohort study consecutively included children without HIV infection who were hospitalized and diagnosed with PJP at West China Second University Hospital, Sichuan University, between February 2022 and June 2025. The patient selection and grouping process is shown in **Figure 1**.

**Figure 1:**
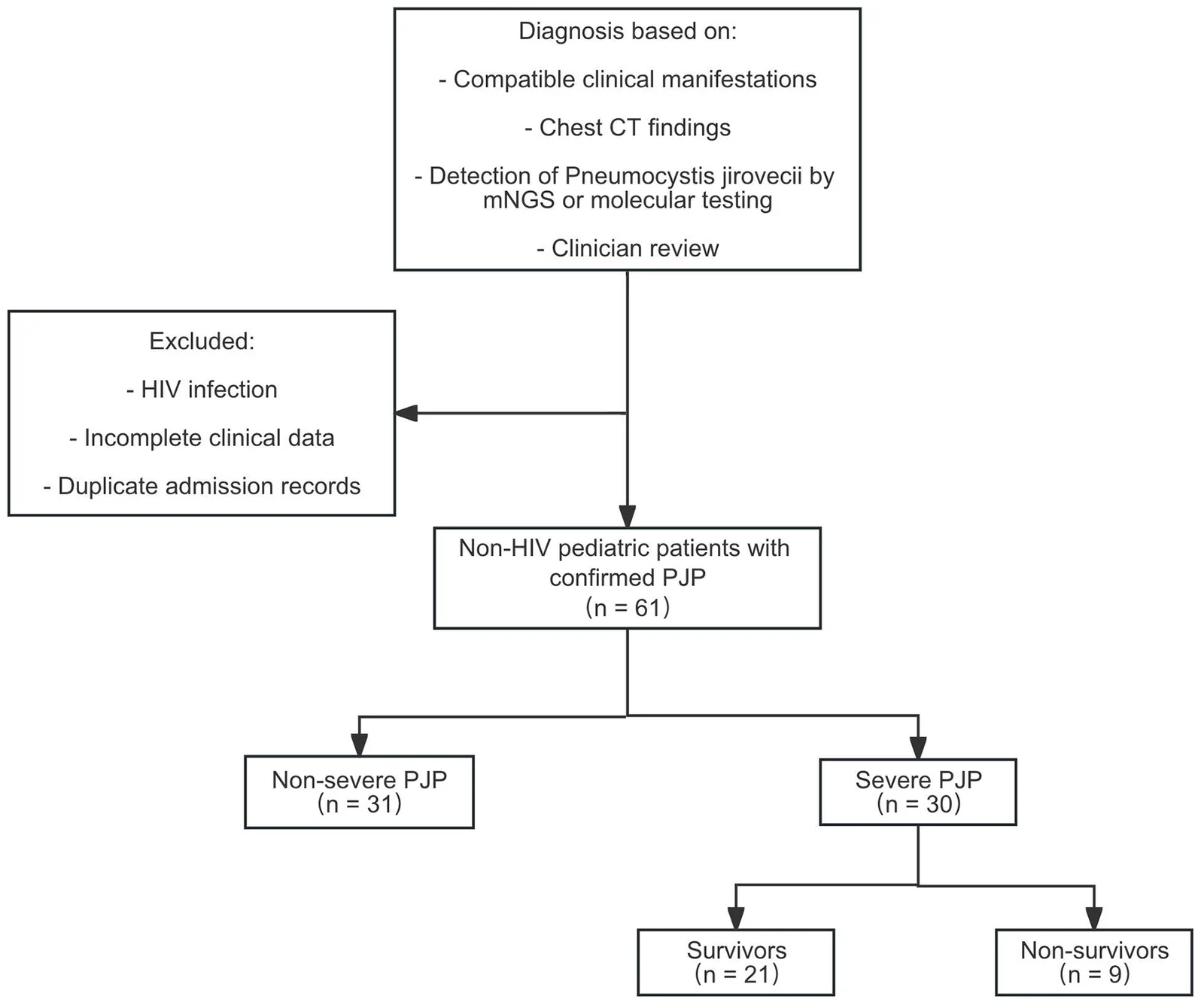
Flow diagram of patient enrollment and study grouping. PJP, *Pneumocystis jirovecii* pneumonia; HIV, human immunodeficiency virus; mNGS, metagenomic next-generation sequencing.

The inclusion criteria were as follows: (1) age <18 years; (2) clinical manifestations suggestive of pulmonary infection, such as fever, cough, or dyspnea; (3) newly developed ground-glass opacities, patchy infiltrates, consolidation, or other pulmonary abnormalities consistent with PJP on chest computed tomography (CT); (4) detection of *P. jirovecii* nucleic acid in respiratory specimens using molecular diagnostic methods; and (5) complete clinical data and in-hospital outcome records. The exclusion criteria were as follows: (1) positive HIV antibody or HIV nucleic acid test; (2) detection of *P. jirovecii* without corresponding clinical or radiological evidence and classification as respiratory tract colonization after comprehensive assessment; (3) the presence of another condition that could fully explain the pulmonary manifestations, without evidence supporting active PJP; and (4) insufficient data for severity classification or missing in-hospital outcome records. For children hospitalized more than once during the study period, only the first hospitalization meeting the inclusion criteria was retained.

The diagnosis of PJP was independently reviewed by two physicians experienced in the diagnosis and treatment of pediatric respiratory diseases. The diagnosis was determined by integrating host susceptibility factors, clinical manifestations, chest imaging findings, microbiological results from respiratory specimens, and findings for other pathogens, with the response to anti-PJP treatment considered when necessary. Disagreements between the two physicians were adjudicated by a third senior physician to reach a consensus.

This study was approved by the Medical Ethics Committee of West China Second University Hospital, Sichuan University (Medical Research 2025, No. 363). Owing to the retrospective study design, the requirement for informed consent from the children and their guardians was waived by the ethics committee.

### 2.2 Data collection

Demographic characteristics, underlying conditions, clinical manifestations, laboratory and immunological parameters, microbiological findings, chest imaging features, treatments, and in-hospital outcomes were extracted from the electronic medical record system. Demographic data included age and sex. Underlying conditions included chronic lung disease, malnutrition, primary immunodeficiency, malignancy, neurometabolic disorders, and previous immunosuppressive therapy. Clinical manifestations included fever, cough, and dyspnea.

Laboratory and immunological parameters included complete blood counts, serum albumin, C-reactive protein (CRP), procalcitonin (PCT), lactate dehydrogenase (LDH), the CD4^+^/CD8^+^ T-cell ratio, and the ratio of arterial oxygen partial pressure to the fraction of inspired oxygen (PaO_2_/FiO_2_). For these parameters, the measurement obtained closest to the time of PJP diagnosis within the 48-h period before or after diagnosis was used. The PaO_2_/FiO_2_ ratio was calculated using contemporaneous arterial blood gas results and the corresponding fraction of inspired oxygen.

Microbiological data included the results of metagenomic next-generation sequencing (mNGS) of bronchoalveolar lavage fluid (BALF), airway secretion cultures, respiratory pathogen nucleic acid tests, antigen tests, and serological tests. Bacteria, viruses, and atypical pathogens other than *P. jirovecii* were recorded. Chest imaging data were obtained from the chest CT examination performed closest to the time of PJP diagnosis. Pulmonary imaging features were recorded, and the total severity score (TSS) was calculated based on chest CT findings. Treatment data included trimethoprim–sulfamethoxazole (TMP–SMX) therapy, intravenous systemic corticosteroid therapy, and invasive mechanical ventilation. In-hospital outcomes included length of hospital stay and in-hospital mortality.

### 2.3 Definitions

#### 2.3.1 Severe PJP

Severe PJP was defined according to criteria for severe pneumonia in the relevant pediatric community-acquired pneumonia guideline (Subspecialty Group of Respiratory, the Society of Pediatrics, Chinese Medical Association et al., 2024). Children meeting any of the following criteria were classified as having severe PJP: poor general condition; feeding refusal or signs of dehydration; impaired consciousness; markedly increased respiratory rate; cyanosis; dyspnea; peripheral oxygen saturation (SpO_2_) ≤92% at rest at sea level or <90% at high altitude; multilobar involvement or involvement of at least two-thirds of a single lobe on chest imaging; pleural effusion; or extrapulmonary complications. Children who did not meet any of these criteria were classified as having non-severe PJP.

#### 2.3.2 Coinfection

Coinfection was retrospectively assessed according to uniform criteria and was defined as the concurrent presence of a clinically significant pathogen other than *P. jirovecii* that met the following conditions: (1) detection of the corresponding pathogen by BALF mNGS, with supplementary evidence from culture, nucleic acid testing, antigen testing, or serological testing when necessary; (2) consistency of the detected pathogen with the child’s clinical manifestations, inflammatory markers, and chest imaging findings; and (3) classification of the organism as a recognized respiratory pathogen. A positive microbiological result without corresponding clinical evidence was not classified as coinfection. *Candida* species, coagulase-negative *Staphylococcus* species, and *Enterococcus* species were generally considered respiratory tract colonizers or contaminants and were not classified as coinfections unless there was clear evidence of invasive infection.

#### 2.3.3 TSS

The TSS was used to evaluate the extent of pulmonary involvement. The lungs were divided into five lobes, and each lobe was scored according to the proportion affected: 0 for no involvement, 1 for <5%, 2 for 5%–25%, 3 for 26%–49%, 4 for 50%–75%, and 5 for >75%. The scores for the five lobes were summed to obtain a total TSS ranging from 0 to 25 (Li et al., 2020). The chest CT examination performed closest to the time of PJP diagnosis was used for scoring. Two radiologists independently assessed the CT images while blinded to the clinical group and outcome. Disagreements were resolved by consensus.

#### 2.3.4 PJP-related systemic corticosteroid therapy

PJP-related systemic corticosteroid therapy was defined as the initiation of intravenous systemic corticosteroids after PJP diagnosis to control PJP-related inflammation, hypoxemia, or respiratory failure. For children already receiving corticosteroids for an underlying condition, treatment was classified as PJP-related corticosteroid therapy only when the dose was substantially increased after PJP diagnosis specifically to control PJP-related pulmonary inflammation or respiratory failure. Continuation of the existing corticosteroid regimen for the underlying condition was not classified as PJP-related corticosteroid therapy.

### 2.4 Statistical analysis

Statistical analyses were performed using SPSS version 29.0 and Python. Continuous variables are presented as medians and interquartile ranges [M (IQR)] and were compared between groups using the Mann–Whitney U test. Categorical variables are presented as numbers and percentages [n (%)] and were compared using Fisher’s exact test. Univariable logistic regression was performed to identify factors associated with severe PJP. Variables for the multivariable models were selected on the basis of clinical relevance, collinearity, and sample size, and multivariable analyses were performed using Firth penalized logistic regression.

Receiver operating characteristic curves were used to evaluate the ability of relevant indicators and combined models to discriminate severe PJP. The 95% confidence intervals for the areas under the curve and comparisons between curves were calculated using the DeLong method, and optimal cutoff values were determined using the Youden index. Among children with severe PJP, clinical characteristics were compared between survivors and non-survivors, and Firth logistic regression with a restricted number of covariates was used to explore factors associated with in-hospital mortality. For Firth regression, 95% confidence intervals were calculated using profile penalized likelihood, and P values were obtained using penalized likelihood ratio tests. All tests were two-sided, and P<0.05 was considered statistically significant.

## 3 Results

### 3.1 Clinical characteristics and comparison according to disease severity

A total of 61 children with non-HIV-associated PJP were included, of whom 30 (49.2%) had severe PJP and 9 (14.8%) died during hospitalization. All nine deaths occurred in the severe group, resulting in an in-hospital mortality rate of 30.0% among children with severe PJP. The median age was 5.7 months (IQR, 3.7–18.0), and 40 patients (65.6%) were male. Cough, dyspnea, and fever were the most common clinical manifestations. The most common underlying conditions or related clinical states included chronic lung disease, malnutrition, malignancy, immunosuppressive therapy, and primary immunodeficiency.

Compared with the non-severe group, the severe group had higher proportions of primary immunodeficiency, fever, dyspnea, and CMV coinfection; higher CRP, PCT, LDH, and TSS values; and lower albumin levels, PaO_2_/FiO_2_ ratios, and CD4^+^/CD8^+^ T-cell ratios (all P<0.05). The severe group also had a longer hospital stay and higher rates of invasive mechanical ventilation and in-hospital mortality. The clinical characteristics and in-hospital outcomes of the two groups are presented in **Table 1**.

**Table 1:** Clinical characteristics and in-hospital outcomes of children with non-HIV-associated PJP according to disease severity.

Variable	Non-severe PJP (n=31)	Severe PJP (n=30)	Total (n=61)	P value
Demographics				
Age, months (M, IQR)	6.8 (3.8-16.0)	5.0 (3.7-29.8)	5.7 (3.7-18.0)	0.994
Male sex, n (%)	21 (67.7%)	19 (63.3%)	40 (65.6%)	0.791
Underlying conditions, n (%)				
Malnutrition	10 (32.3%)	15 (50.0%)	25 (41.0%)	0.198
Chronic lung disease	18 (58.1%)	15 (50.0%)	33 (54.1%)	0.611
Primary immunodeficiency	2 (6.5%)	8 (26.7%)	10 (16.4%)	0.043
Malignancy	9 (29.0%)	6 (20.0%)	15 (24.6%)	0.554
Neurometabolic disorder	1 (3.2%)	3 (10.0%)	4 (6.6%)	0.354
Immunosuppressive therapy	6 (19.4%)	6 (20.0%)	12 (19.7%)	1.000
Clinical manifestations, n (%)				
Fever	13 (41.9%)	22 (73.3%)	35 (57.4%)	0.020
Cough	31 (100.0%)	28 (93.3%)	59 (96.7%)	0.238
Dyspnea	24 (77.4%)	30 (100.0%)	54 (88.5%)	0.011
Wheezing	15 (48.4%)	8 (26.7%)	23 (37.7%)	0.114
Laboratory findings				
D-dimer, mg/L (M, IQR)	1.0 (0.7–1.7)	2.0 (1.4–3.2)	1.6 (0.8–2.5)	0.013
Lactate, mmol/L (M, IQR)	1.1 (0.8-1.3)	1.1 (0.9-1.6)	1.1 (0.8-1.6)	0.434
WBC count, ×10^9^/L (M, IQR)	9.4 (6.9-15.6)	13.3 (3.3-17.8)	10.4 (5.7-17.5)	0.846
Neutrophil count, ×10^9^/L (M, IQR)	2.7 (1.8-7.1)	5.4 (2.1-11.7)	3.2 (1.8-9.4)	0.286
Lymphocyte count, ×10^9^/L (M, IQR)	4.6 (2.8-7.0)	2.3 (0.9-7.2)	3.4 (1.9-7.0)	0.105
Platelet count, ×10^9^/L (M, IQR)	390.0 (317.5-461.5)	387.5 (271.8-469.8)	390.0 (305.0-469.0)	0.971
Albumin, g/L (M, IQR)	40.2 (39.1–43.7)	38.2 (32.1–40.2)	39.7 (36.2–42.0)	0.024
CRP, mg/L (M, IQR)	1.3 (0.5-15.6)	25.9 (1.4-61.2)	3.2 (0.8-42.7)	0.006
PCT, ng/mL (M, IQR)	0.2 (0.1-0.3)	0.7 (0.2-6.7)	0.3 (0.2-0.8)	<0.001
LDH, U/L (M, IQR)	335(286–450)	646(489–827)	478.0 (323–667)	<0.001
CD4+/CD8+ T-cell ratio (M, IQR)	2.20 (1.35–2.70)	1.25 (0.75–2.08)	1.80 (1.10–2.40)	0.002
Respiratory parameters				
PaO_2_/FiO_2_ ratio (M, IQR)	187.2 (155.0-251.2)	122.0 (74.0-153.0)	155.0 (121.0-188.7)	<0.001
Radiological findings				
TSS (M, IQR)	7.0 (5.0-9.5)	10.0 (8.0-14.0)	8.0 (6.0-12.0)	0.003
Coinfections, n (%)				
CMV	3 (9.7%)	13 (43.3%)	16 (26.2%)	0.004
Bacteria	7 (22.6%)	7 (23.3%)	14 (23.0%)	1.000
Atypical pathogen	2 (6.5%)	3 (10.0%)	5 (8.2%)	0.671
Treatment and outcomes				
Intravenous systemic corticosteroid use, n (%)	2 (6.5%)	6 (20.0%)	8 (13.1%)	0.147
TMP-SMX duration, days (M, IQR)	8.0 (4.5–13.5)	18.5 (7.2–21.0)	13.0 (5.0–21.0)	0.011
Invasive ventilation, n (%)	0 (0%)	15 (50.0%)	15 (24.6%)	<0.001
Length of hospital stay, days (M, IQR)	18.0 (11.5–22.5)	26.5 (19.8–39.8)	22.0 (15.0–31.0)	<0.001
In-hospital mortality, n (%)	0 (0%)	9 (30.0%)	9 (14.8%)	<0.001

CI, confidence interval; CMV, cytomegalovirus; CRP, C-reactive protein; IQR, interquartile range; LDH, lactate dehydrogenase; M, median; PCT, procalcitonin; PJP, *Pneumocystis jirovecii* pneumonia; TMP–SMX, trimethoprim–sulfamethoxazole; TSS, CT severity score; WBC, white blood cell; FiO_2_, fraction of inspired oxygen; PaO_2_, partial pressure of arterial oxygen.

### 3.2 Factors associated with severe PJP

Univariable logistic regression analysis showed that primary immunodeficiency, fever, decreased albumin, elevated PCT and LDH, decreased CD4^+^/CD8^+^ T-cell and PaO_2_/FiO_2_ ratios, elevated TSS, and CMV coinfection were associated with severe PJP (**Table 2**).

**Table 2:** Univariable logistic regression analysis of factors associated with severe PJP.

Variable	OR	95% CI	P value
Age, per month	1.007	0.995–1.020	0.247
Male sex	0.823	0.286–2.368	0.717
Malnutrition	2.100	0.743–5.935	0.162
Chronic lung disease	0.722	0.263–1.984	0.528
Primary immunodeficiency	5.273	1.017–27.335	0.048
Malignancy	0.611	0.187–1.997	0.415
Neurometabolic disorder	3.333	0.327–33.993	0.310
Immunosuppressive therapy	1.042	0.295–3.682	0.949
Fever	3.808	1.295–11.199	0.015
Wheezing	0.388	0.133–1.134	0.084
D-dimer, per 1 mg/L	1.262	0.957–1.664	0.099
Lactate, mmol/L	1.696	0.826–3.484	0.150
WBC, ×10^9^/L	1.018	0.949–1.091	0.622
Neutrophil count, ×10^9^/L	1.060	0.960–1.170	0.252
Lymphocyte count, ×10^9^/L	0.926	0.797–1.074	0.309
Platelet count, ×10^9^/L	1.000	0.997–1.003	0.990
Albumin, per 1 g/L	0.904	0.818–0.998	0.046
CRP, per 1 mg/L	1.011	0.998–1.024	0.098
PCT, per 1 ng/mL	1.294	1.037–1.614	0.022
LDH, per 100 U/L	1.836	1.290–2.613	<0.001
CD4^+^/CD8^+^ T-cell ratio	0.519	0.288–0.937	0.030
PaO_2_/FiO_2_, per 10-unit increase	0.732	0.615–0.870	<0.001
TSS, per 1-point increase	1.244	1.064–1.454	0.006
CMV coinfection	7.137	1.773–28.728	0.006
Bacterial coinfection	1.043	0.316–3.442	0.944
Atypical pathogen coinfection	1.611	0.250–10.395	0.616

ORs for continuous variables represent the change in the odds of severe PJP per 1-unit increase, except for LDH and PaO_2_/FiO_2_, which are reported per 100-U/L and 10-unit increases, respectively. For binary variables, the reference categories were female sex and the absence of the corresponding condition, symptom, or coinfection. Variables that directly reflected or overlapped with the definition of severe pneumonia were evaluated in the univariable analysis but were not considered for inclusion in the multivariable models. Cough and dyspnea were not included in the univariable logistic regression analysis because of sparse data or quasi-complete separation; dyspnea also overlapped with the definition of severe pneumonia.

CI, confidence interval; CMV, cytomegalovirus; CRP, C-reactive protein; LDH, lactate dehydrogenase; OR, odds ratio; PCT, procalcitonin; PJP, *Pneumocystis jirovecii* pneumonia; TSS, CT severity score; WBC, white blood cell.

In the primary model (Model 1), multivariable Firth logistic regression analysis showed that elevated LDH and CMV coinfection remained associated with severe PJP after adjustment for PCT and the CD4^+^/CD8^+^ T-cell ratio. For every 100-U/L increase in LDH, the odds of severe PJP increased by 53.2% (adjusted odds ratio [aOR]=1.532, 95% confidence interval [CI]: 1.061–2.374; P = 0.021). Children with CMV coinfection had 5.164-fold higher odds of severe PJP than those without CMV coinfection (aOR=5.164, 95% CI: 1.099–28.048; P = 0.037). PCT and the CD4^+^/CD8^+^ T-cell ratio did not reach statistical significance after adjustment for the other variables.

In the sensitivity analysis model incorporating LDH, albumin, primary immunodeficiency, and CMV coinfection (Model 2), LDH remained associated with severe PJP (per 100-U/L increase: aOR=1.801, 95% CI: 1.288–2.771; P<0.001), whereas albumin, primary immunodeficiency, and CMV coinfection did not reach statistical significance. These findings indicate that the association between LDH and severe PJP was relatively consistent across different model specifications, whereas the effect estimate for CMV coinfection was sensitive to covariate selection. The multivariable analysis results are presented in **Table 3**.

**Table 3:** Multivariable Firth penalized logistic regression analyses of factors associated with severe PJP.

Variables	aOR	95% CI	P value
Model 1			
LDH, per 100 U/L increase	1.532	1.061–2.374	0.021
PCT, per 1 ng/mL increase	1.207	0.987–1.580	0.067
CD4^+^/CD8^+^ T-cell ratio	0.768	0.391–1.471	0.415
CMV coinfection	5.164	1.099–28.048	0.037
Model 2			
LDH, per 100 U/L increase	1.801	1.288–2.771	<0.001
Albumin, per 1 g/L increase	1.018	0.897–1.168	0.762
Primary immunodeficiency	4.334	0.772–30.991	0.095
CMV coinfection	2.940	0.531–18.807	0.213

Values are presented as adjusted odds ratios (aORs) with 95% confidence intervals (CIs). Model 1 was the primary model and included LDH, PCT, the CD4^+^/CD8^+^ T-cell ratio, and CMV coinfection. Model 2 was the sensitivity analysis model and included LDH, albumin, primary immunodeficiency, and CMV coinfection. The 95% CIs were calculated using profile penalized likelihood, and P values were obtained using penalized likelihood ratio tests.

CI, confidence interval; CMV, cytomegalovirus; LDH, lactate dehydrogenase; PCT, procalcitonin; PJP, *Pneumocystis jirovecii* pneumonia.

### 3.3 Discriminative performance of LDH and CMV coinfection for severe PJP

Receiver operating characteristic curve analysis showed that the AUC of LDH for discriminating severe from non-severe PJP was 0.868 (95% CI: 0.776–0.960). Based on the maximum Youden index, the optimal LDH cutoff was 423.0 U/L (Youden index=0.610), with a sensitivity of 90.0% and a specificity of 71.0%.

The AUC of CMV coinfection alone for discriminating severe from non-severe PJP was 0.668 (95% CI: 0.564–0.773). Using the presence of CMV coinfection as the classification criterion, the sensitivity was 43.3% and the specificity was 90.3%.

The model combining LDH and CMV coinfection had an AUC of 0.878 (95% CI: 0.791–0.966). Based on the maximum Youden index, the optimal predicted probability cutoff for the combined model was 0.376, with a sensitivity of 86.7% and a specificity of 74.2%. Compared with LDH alone, the AUC of the combined model increased by 0.011, but the difference was not statistically significant (DeLong test, P = 0.701), indicating that the addition of CMV coinfection did not significantly improve the discriminative performance of LDH for severe PJP. The ROC curves are shown in **Figure 2**.

**Figure 2:**
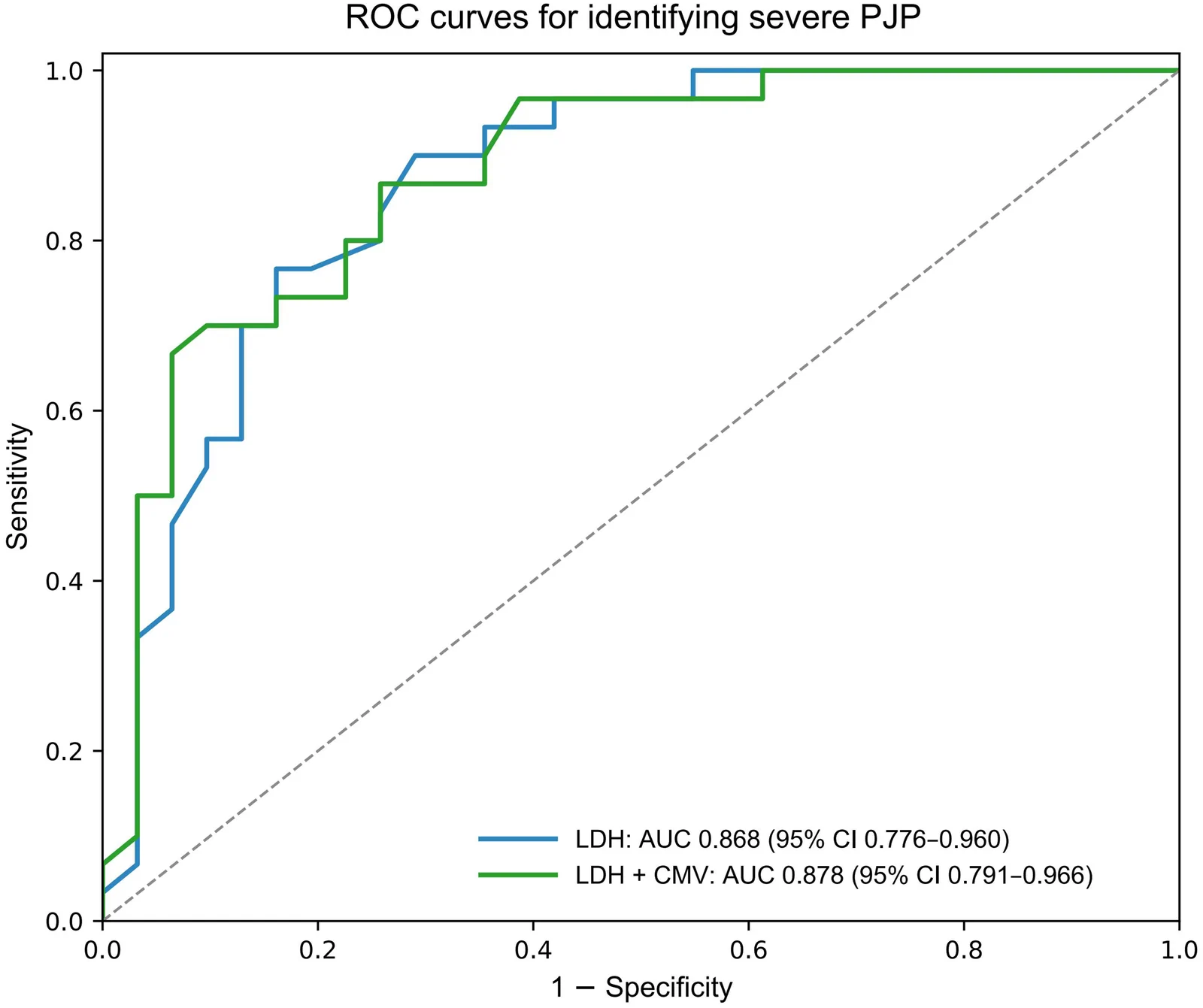
Receiver operating characteristic curves of LDH alone and in combination with CMV co-infection for identifying severe PJP. The diagonal dashed line represents the reference line. AUC, area under the curve; CI, confidence interval; CMV, cytomegalovirus; LDH, lactate dehydrogenase; PJP, *Pneumocystis jirovecii* pneumonia; ROC, receiver operating characteristic.

### 3.4 Comparison of clinical characteristics between survivors and non-survivors with severe PJP

Among the 30 children with severe PJP, 21 survived and 9 died during hospitalization. Compared with survivors, non-survivors had higher proportions of malnutrition and CMV coinfection, higher LDH and PCT levels, and lower albumin levels, PaO_2_/FiO_2_ ratios, and CD4^+^/CD8^+^ T-cell ratios (all P<0.05). The clinical characteristics of the two groups are presented in **Table 4**.

**Table 4:** Comparison of clinical characteristics between survivors and non-survivors among children with severe PJP.

Variable	Survivors (n=21)	Non-survivors (n=9)	P value
Demographics			
Age, months (M, IQR)	5 (3.93–36)	4.03 (3.67–7)	0.469
Male sex, n (%)	13 (61.9%)	6 (66.7%)	1.000
Underlying conditions, n (%)			
Malnutrition	7 (33.3%)	8 (88.9%)	0.014
Chronic lung disease	10 (47.6%)	5 (55.6%)	1.000
Primary immunodeficiency	6 (28.6%)	2 (22.2%)	1.000
Malignancy	4 (19.0%)	2 (22.2%)	1.000
Neurometabolic disorder	1 (4.8%)	2 (22.2%)	0.207
Immunosuppressive therapy	3 (14.3%)	3 (33.3%)	0.329
Clinical manifestations, n (%)			
Fever	15 (71.4%)	7 (77.8%)	1.000
Cough	19 (90.5%)	9 (100.0%)	1.000
Dyspnea	21 (100.0%)	9 (100.0%)	—
Wheezing	6 (28.6%)	2 (22.2%)	1.000
Laboratory findings			
D-dimer, mg/L (M, IQR)	2.12 (1.47–3.64)	1.97 (1.12–2.46)	0.455
Lactate, mmol/L (M, IQR)	1.1 (0.9–1.6)	1.1 (0.9–1.65)	0.855
WBC count, ×10^9^/L (M, IQR)	16.1 (8.1–17.9)	5.6 (2.5–13.2)	0.118
Neutrophil count, ×10^9^/L (M, IQR)	7.02 (3.53–11.9)	2.11 (0.82–4.85)	0.046
Lymphocyte count, ×10^9^/L (M, IQR)	2.32 (0.84–7.4)	2.25 (0.95–3.98)	0.734
Platelet count, ×10^9^/L (M, IQR)	377 (264–439)	428 (331–514)	0.483
Albumin, g/L (M, IQR)	39.8 (37.9–42)	31.7 (30.9–32.3)	<0.001
CRP, mg/L (M, IQR)	11.4 (1.4–55.8)	44.1 (1.3–71.8)	0.468
PCT, ng/mL (M, IQR)	0.34 (0.16–0.76)	2.95 (1.4–11.1)	0.007
LDH, U/L (M, IQR)	498 (465–667)	864 (785–1076)	<0.001
Immunological findings			
CD4^+^/CD8^+^ T-cell ratio (M, IQR)	1.6 (1.1–2.2)	0.9 (0.6–1)	0.011
Respiratory parameters			
PaO_2_/FiO_2_ ratio (M, IQR)	152 (121–155)	67.9 (63.1–68)	<0.001
Radiological findings			
TSS	10 (7–13)	12 (9–14)	0.328
Coinfections, n (%)			
CMV coinfection	6 (28.6%)	7 (77.8%)	0.020
Bacterial coinfection	5 (23.8%)	2 (22.2%)	1.000
Atypical pathogen coinfection	3 (14.3%)	0 (0.0%)	0.534

Data are presented as median (IQR) or n (%). Continuous variables were compared using the Mann–Whitney U test, and categorical variables were compared using Fisher’s exact test. The P value for dyspnea was not estimable because all children in both groups had dyspnea.

CMV, cytomegalovirus; CRP, C-reactive protein; FiO_2_, fraction of inspired oxygen; IQR, interquartile range; LDH, lactate dehydrogenase; PaO_2_, partial pressure of arterial oxygen; PCT, procalcitonin; PJP, *Pneumocystis jirovecii* pneumonia; TSS, CT severity score; WBC, white blood cell.

### 3.5 Exploratory analysis of factors associated with in-hospital mortality among children with severe PJP

Because only nine in-hospital deaths occurred in the severe PJP group, the exploratory multivariable Firth logistic regression model included only LDH and albumin to reduce the risk of model overfitting while maintaining clinical relevance and model parsimony. After mutual adjustment, every 100-U/L increase in LDH was associated with a 65.4% increase in the odds of in-hospital mortality (aOR=1.654, 95% CI: 1.080–4.271; P = 0.018), whereas every 1-g/L increase in albumin was associated with a 39.5% decrease in the odds of in-hospital mortality (aOR=0.605, 95% CI: 0.251–0.878; P = 0.004). The exploratory multivariable analysis results are presented in **Table 5**.

**Table 5:** Exploratory multivariable Firth logistic regression analysis of factors associated with in-hospital mortality among children with severe PJP.

Variable	aOR	95% CI	P value
LDH, per 100 U/L increase	1.654	1.080–4.271	0.018
Albumin, per 1 g/L increase	0.605	0.251–0.878	0.004

Values are presented as adjusted odds ratios (aORs) with 95% confidence intervals (CIs). Given the limited number of in-hospital deaths, the exploratory model simultaneously included only LDH and albumin. The 95% CIs were calculated using profile penalized likelihood, and P values were obtained using penalized likelihood ratio tests.

aOR, adjusted odds ratio; CI, confidence interval; LDH, lactate dehydrogenase; PJP, *Pneumocystis jirovecii* pneumonia.

## 4 Discussion

This study included 61 children with non-HIV-associated PJP and systematically compared the clinical characteristics of patients with severe and non-severe disease. Factors associated with severe PJP and in-hospital mortality were further explored. Nearly half of the patients in this cohort developed severe PJP. All nine in-hospital deaths occurred in the severe group, corresponding to an overall in-hospital mortality rate of 14.8% and an in-hospital mortality rate of 30.0% among patients with severe PJP. In previous studies, Ling et al. reported an overall mortality rate of 41.7% among 60 children with non-HIV-associated PJP, whereas a retrospective study by Kim et al. reported a 60-day mortality rate of 35.7% among 14 children. Another single-center pediatric study spanning 30 years reported a 30-day mortality rate of 20.0% overall and 31.25% among children without HIV infection (Kim et al., 2016; Ling et al., 2018; García-Moreno et al., 2020). In a cohort of hospitalized children aged <3 years who tested positive for *P. jirovecii*, the overall mortality rate was 18.8%; however, that study included both active infection and colonization (Menu et al., 2021). More recently, a study of 40 critically ill children with non-HIV-associated PJP admitted to a pediatric intensive care unit (PICU) reported 30- and 90-day all-cause mortality rates of 12.5% and 22.5%, respectively (An et al., 2025). Our findings further demonstrate that non-HIV-associated PJP in children remains associated with a substantial burden of severe illness and mortality. Variations in mortality across studies may be attributable to differences in the distribution of underlying diseases, degree of immunosuppression, diagnostic and severity criteria for PJP, and duration of outcome assessment. For example, cellular immune defects in some primary immunodeficiencies are not readily reversible, unlike immune dysfunction that may improve following reduction of immunosuppressive therapy or effective antiretroviral treatment. This limited potential for immune recovery may contribute to poorer outcomes.

LDH was the most consistent marker associated with severe disease in this study. Elevated LDH was associated with severe PJP in both the primary and sensitivity analysis models and was also associated with in-hospital mortality in the exploratory mortality model among children with severe PJP. Previous meta-analyses of adults with non-HIV-associated PJP similarly demonstrated an association between elevated LDH and an increased risk of mortality (Liu et al., 2017; Wang et al., 2021). A recent adult cohort study also identified LDH as an independent factor associated with severe PJP and reported an optimal cutoff value of 399.5 U/L (Li et al., 2026), which was close to the cutoff of 423.0 U/L identified in our study. The AUC of LDH was 0.868, indicating good discrimination between severe and non-severe PJP. LDH is widely distributed across multiple tissues, and elevated levels are more likely to reflect the combined effects of pulmonary inflammation, tissue injury, and overall disease burden than to represent a PJP-specific biomarker (Sun et al., 2021). Accordingly, LDH alone has limited specificity for identifying PJP. Moreover, the cutoff value and its discriminative performance were derived from the same cohort without internal resampling-based correction or external validation. Therefore, 423.0 U/L may serve as a reference for early risk identification but should not be used as a standalone threshold for diagnosis or treatment decisions. Future studies should also account for age-specific pediatric reference ranges and validate this cutoff in multicenter cohorts.

CMV coinfection was associated with severe PJP in the primary model, broadly consistent with previous adult cohorts of non-HIV-associated PJP showing associations between CMV coinfection and disease severity or adverse outcomes (Yu et al., 2017; Zou et al., 2022; Perret et al., 2024). Biologically, PJP and CMV coinfection may reflect more profound impairment of host cell-mediated immunity. In addition, CMV can promote immune evasion by interfering with antigen presentation and T-cell responses (Patro, 2019), and CMV-related inflammation and pulmonary tissue injury may compound the lung injury caused by PJP. However, the 95% CI for CMV coinfection was wide in the present study, and the association did not reach statistical significance in the sensitivity analysis model. Furthermore, adding CMV coinfection to LDH increased the AUC by only 0.011, and the difference was not statistically significant. These findings indicate that the estimated effect of CMV coinfection was sensitive to covariate selection and that its incremental discriminative value beyond LDH remains uncertain. Nevertheless, CMV coinfection may still provide a clinical indication of host immune vulnerability and the risk of severe disease and should be evaluated in conjunction with the host immune status, viral load and its dynamic changes, and evidence of end-organ involvement. Because this study did not evaluate the effectiveness of anti-CMV therapy, its findings cannot be used to establish indications for antiviral treatment. Relevant monitoring parameters, treatment thresholds, and the clinical benefits of antiviral therapy require further investigation in prospective studies.

Among children with severe PJP, malnutrition and CMV coinfection were more frequent in non-survivors, who also had higher LDH and PCT levels and lower albumin levels, neutrophil counts, PaO_2_/FiO_2_ ratios, and CD4^+^/CD8^+^ T-cell ratios. These differences may reflect more extensive pulmonary tissue injury, systemic inflammation, and impaired oxygenation among non-survivors, accompanied by reduced nutritional reserves and impaired immune function. In the exploratory Firth regression model that included only LDH and albumin, elevated LDH and decreased albumin were both associated with in-hospital mortality. Previous studies of children with severe community-acquired pneumonia and PICU cohorts have also associated low albumin levels with adverse outcomes or an increased risk of mortality (Kittisakmontri et al., 2016; Shi et al., 2020). During severe infection, decreased albumin levels may result from multiple factors, including systemic inflammation, increased capillary permeability, reduced synthesis, and poor nutritional status. Albumin may therefore represent an integrated marker of host nutritional reserves and overall disease burden, and the observed association should not be interpreted as evidence that albumin itself exerts a protective effect.

Beyond the markers evaluated in our cohort, β-D-glucan (BDG) and quantitative *P. jirovecii* burden may also have clinical value. A retrospective PICU study including 36 children with PJP and 61 without PJP, all HIV-negative, reported a diagnostic sensitivity of 57.6% for BDG (Zhang et al., 2024), whereas an adult non-HIV PJP study found no significant difference in baseline BDG levels between survivors and non-survivors (Matsumura et al., 2011), suggesting that BDG may aid in PJP diagnosis while its value for assessing disease severity and prognosis remains uncertain. Higher *P. jirovecii* burden measured by quantitative or digital PCR has also been associated with more severe disease, impaired oxygenation, and increased mortality in non-HIV PJP (Bian et al., 2023; Grønseth et al., 2023), supporting its potential as a complementary marker for risk stratification. However, incomplete BDG data and the lack of standardized quantitative fungal-burden measurements in our cohort precluded further evaluation of their associations with disease severity and prognosis. Future studies using systematic BDG testing and standardized quantification of *P. jirovecii* burden are needed to clarify their roles in severity assessment and prognosis in pediatric non-HIV PJP.

This study has several limitations. First, it was a single-center retrospective study with a relatively small sample and a limited number of in-hospital deaths. Although Firth regression was used to reduce estimation bias arising from the small sample and sparse data, the risks of overfitting, residual confounding, and unstable effect estimates could not be eliminated. In addition, ROC performance was evaluated within the derivation cohort without internal resampling-based correction or external validation, which may have resulted in overestimation of the actual discriminative performance. Second, PJP and coinfections were adjudicated by integrating clinical, radiological, and microbiological evidence. Despite the use of standardized criteria and review by multiple physicians, the absence of standardized quantitative measures of *P. jirovecii* burden limited our ability to use organism load as an additional discriminator between active PJP and *P. jirovecii* colonization in children whose clinical and radiological manifestations might instead be attributable to concomitant respiratory infections. Therefore, residual misclassification cannot be completely excluded. Finally, this study did not systematically evaluate BDG levels, the association between *P. jirovecii* burden and disease severity or prognosis, CMV viral load and its dynamic changes, or the effects of the timing of anti-PJP and anti-CMV treatment on clinical outcomes.

## 5 Conclusion

In conclusion, non-HIV-associated PJP in children was associated with high rates of severe disease and in-hospital mortality. The association between elevated LDH and severe PJP was consistent across different models, and LDH showed good discrimination for severe disease. CMV coinfection was associated with severe PJP in the primary model, but its effect estimate was sensitive to model specification, and its addition did not significantly improve the discriminative performance of LDH. Exploratory analyses further suggested that elevated LDH and decreased albumin may be associated with in-hospital mortality among children with severe PJP. These findings provide preliminary evidence for early risk stratification in children with non-HIV-associated PJP but require validation in larger prospective multicenter studies.

## Data Availability

The original contributions presented in the study are included in the article/**Supplementary Material**. Further inquiries can be directed to the corresponding author.
